# Electroacupuncture Suppresses CCI-Induced Neuropathic Pain through GABAA Receptors

**DOI:** 10.1155/2022/4505934

**Published:** 2022-10-07

**Authors:** Sisi Li, Xia Jiang, Qiaoyun Wu, Yun Jin, Rong He, Jie Hu, Yuyin Zheng

**Affiliations:** ^1^Department of Physical Medicine and Rehabilitation, The Second Affiliated Hospital and Yuying Children's Hospital of Wenzhou Medical University, Wenzhou, Zhejiang, China; ^2^Integrative & Optimized Medicine Research Center, China-USA Institute for Acupuncture and Rehabilitation of Wenzhou Medical University, Wenzhou, Zhejiang, China; ^3^Department of Rehabilitation, Wenzhou Hospital of Integrated Traditional Chinese and Western Medicine, Wenzhou, Zhejiang, China

## Abstract

Neuropathic pain remains a chronic and intractable pain. Recent studies have shown a close relationship between gamma-aminobutyric acid A (GABAA) receptor and neuropathic pain. Spinal cord GABAA receptors are key modulators of pain processing. Electroacupuncture (EA) is currently used worldwide to relieve pain. The immunomodulatory effect of EA in animals has been proposed in previous studies. However, it remains unclear how EA contributes to alleviating neuropathic pain. In this study, the chronic constriction injury (CCI) rat model was used to explore the relationship between GABAA receptor and neuropathic pain. We also investigated whether EA treatment could ameliorate pain hypersensitivity by modulating the GABAA receptor. To determine the function of EA in neurological diseases, in this study, the mechanical withdrawal threshold (MWT) and thermal withdrawal latency (TWL) were assessed to determine the threshold of pain. In addition, we used Western blot, immunofluorescence, and real-time quantitative PCR to confirm whether EA treatment relieves pain hypersensitivity by regulating GABAA receptors. The morphology of synapse was examined using an electron microscope. In the present study, EA relieved mechanical allodynia and thermal hyperalgesia. EA also inhibited microglial activation in the spinal cord, accompanied by increased levels of GABAAR*α*2, GABAAR*α*3, and GABAAR*γ*2 subunits. However, the analgesic effect of EA was attenuated by treatment with the GABAA receptor antagonist bicuculine. Overall, the present results indicate that microglia and GABAA receptor might participate in EA analgesia. These results contribute to our understanding of the impact of EA on rats after sciatic nerve compression, providing a theoretical basis for the clinical application of EA analgesia.

## 1. Introduction

Neuropathic pain is a disorder defined as pain caused by primary lesions and dysfunction of the somatosensory system and characterized by spontaneous pain, hyperalgesia, and allodynia [1]. It is a serious health problem that affects millions of people throughout the world. However, the pathophysiology of the disease remains unknown. Better understanding of the molecular pathogenesis underlying neuropathic pain will undoubtedly contribute to the development of new therapies. Chronic constriction injury (CCI) is a widely used animal model in biomedical research, and it is an accepted model for neuropathic pain [2].

Recently, emerging evidence has suggested that the spinal cord microglia is important regulator of neuropathic pain [3]. In response to neuropathic pain, the immune reaction is activated, with an initial activation of microglia in the spinal cord dorsal horn, ipsilateral to the injury [4]. Gamma-aminobutyric acid (GABA) is an important inhibitory neurotransmitter in the central nervous system (CNS) [5]. Reduced GABA receptor system function will result in epilepsy, sleep disorder, anxiety, spasm, stress, addiction depression, and pain, whereas excessive activation of the GABA receptor will result in schizophrenia [6]. It is widely accepted that there are three major types of GABA receptors: GABAA, GABAB, and GABAC types. GABAA receptors are the major mediators of neural inhibition in the brain among the three GABA receptors. GABAA receptors consist of *α*, *β*, *γ*, *δ*, *ε*, *θ*, *π*, and *ρ* subunits [7]. Although there are many kinds of GABAA receptor subunits, the different composition of GABAA receptor subunits leads to their different functions and pharmacological properties. Spinal GABAA receptor subunits mainly contain *α*2 and *α*3 together with *β*3 subunit and *γ*2 subunit [8]. It has been found that the *α* and *β* subunits constitute the binding region, while the *γ*2 subunit is an essential subunit for the establishment of normal signal transduction [9]. The binding of *α* and *γ* subunits increases the frequency of chloride channel opening to enhance postsynaptic inhibition. In addition, it has been demonstrated that receptor *α*2 and *α*3 subunits play an important role in spinal cord antihyperalgesia induced by benzodiazepines [10]. Chronic pain states are related to the decrease in GABA mediated inhibition [11]. GABAA receptor agonists, such as benzodiazepines and barbiturates, may induce sedation, ameliorate anxiety, and provide analgesia [12]. Neural injuries can result in inhibitory actions of GABA in spinal neurons; enhancing GABA synaptic inhibition has shown to be effective in relieving pain due to a complex series of physiological reactions [13].

Acupuncture is a typical traditional medicine method which has been used in China for thousands of years. Numerous clinical studies and experimental studies have indicated the therapeutic effects of acupuncture in the treatment of pain [14, 15]. Electroacupuncture (EA) is a modified form of combining traditional acupuncture with modern electrotherapy, and it refers to the stimulation of acupoints by applying a pulsating electrical current through acupuncture needles [16, 17]. The therapeutic onset for EA treatment is associated with the production and release of various endogenous bioactive substances, containing monoamines, opioids, neurotrophins, adenosine, and cytokines [18–20]; the precise mechanism underlying the action of EA analgesia remains unknown.

In this study, we aimed to determine whether EA treatment relieves mechanical allodynia and thermal hyperalgesia in CCI rats through regulation of the GABAA receptor. Here, we examined the effect of EA on the activity of microglia. Then, we used GABAA receptor antagonist, bicuculine (BIC), to identify the role of the GABAA receptors in the analgesic effect of EA and the relationship between EA and the GABAA receptor, and their effects on the morphology of synapse were also determined.

## 2. Materials and Methods

### 2.1. Animals

Seventy-five adult male Sprague–Dawley rats (150–200 g) were provided by the Laboratory Animal Center of Wenzhou Medical University. Animals were housed in a room under a temperature-controlled (22°C–24°C) room, with a relative humidity of 45–55%, 12 h light/dark cycle, and free access to food and water. All animal experiments conducted in the current study adhered to the protocols approved by the Institutional Animal Care and Use Committee of Wenzhou Medical University and were performed in accordance with the policies issued by the National Institutes of Health and the International Association for the Study of Pain. All efforts were made to minimize the number of animals used and their suffering.

### 2.2. Chronic Constriction Injury Model

**Figure 1:**
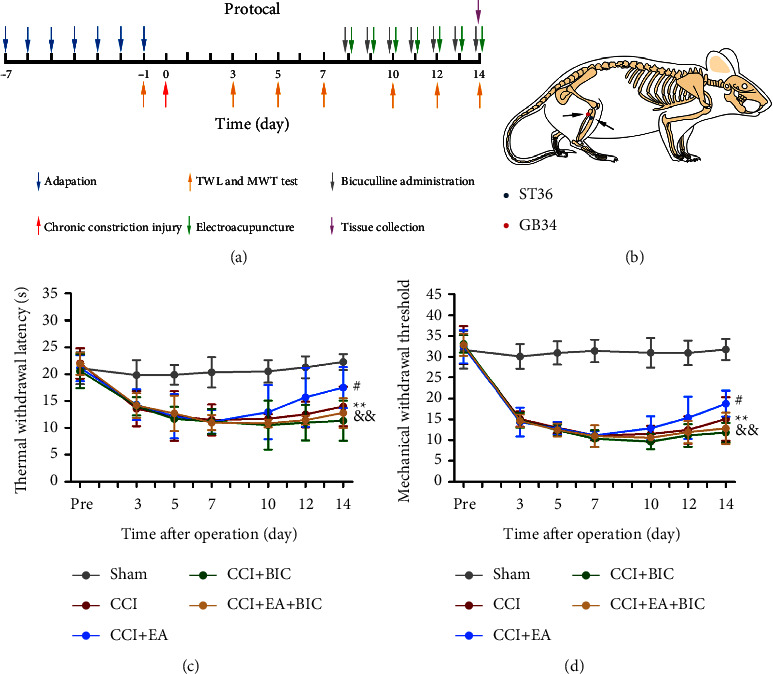
EA relieves mechanical and thermal hyperalgesia in CCI rats through the GABAA receptor. (a) The flow chart of the experiment. (b) Schematic of the locations of the acupoints in rats. (c) Thermal withdrawal latency (TWL). (d) Mechanical withdrawal threshold (MWT). The data are presented as the means ± SD (*n* = 8 per group). ^*∗∗*^*P* < 0.01 compared with the Sham group; ^#^*P* < 0.05 compared with the CCI group; ^&&^*P* < 0.01 compared with the CCI + EA group.

The CCI model of neuropathic pain in rats was induced with the method previously described [21, 22]. After anaesthesia with 2% sodium pentobarbital (30 mg/kg, i.p.), the right sciatic nerve at the mid-thigh level was exposed. The nerve proximal to the trifurcation was subsequently ligated by loosely tying four 4-0 chromic gut sutures with a spacing of approximately 1 mm. The right sciatic nerve in the Sham group was only exposed for 2-3 min but not ligated. All rats were randomly allocated into 5 groups, containing the Sham group, CCI group, CCI plus EA group (CCI + EA), CCI plus BIC group (CCI + BIC), and CCI plus EA + BIC group (CCI + EA + BIC). After BIC was dissolved according to the manufacturers' instructions, it was administered at a dose of 1 mL/kg of animal body weight [23]. The pain threshold was assessed before CCI surgery and at days 3, 5, 7, 10, 12, and 14 after the CCI surgery. Experimental workflow is described in Figure 1(a).

### 2.3. Mechanical Withdrawal Threshold (MWT)

All behavioural tests were performed between 15:00 and 20:00 p.m. by an examiner blinded to the treatment groups. We applied 2392 Electronic von Frey Anesthesiometer (IITC Life Science, California, USA) to calculate MWT to evaluate the behavioural response to mechanical stimulation. Animals were placed individually in a plastic cage (20 cm × 14 cm × 16 cm) with a wire mesh bottom, allowed to adapt to the environment for at least 30 min. The plantar surface of the hind paw was stimulated by using von Frey with a series of increasing stimulations (0.1 to 70 g) until the rat twitched its paw. Maximum values were recorded. Five trials were performed per animal, and 5 min intervals were given between trials. The mean value was considered as the hind paw MWT after excluding the maximum and minimum forces.

### 2.4. Thermal Withdrawal Latency (TWL)

Thermal hyperalgesia was used to evaluate the TWL using the A37370 plantar test apparatus (Ugo-Basile, Milan, Italy). Animals were separated and placed in a transparent, square box (17 cm × 11.5 cm × 14 cm). After a 15 min acclimation period, the radiant heat was set at 60°C, and a radiant heat was applied by aiming a beam of light onto the plantar surface of the hind paw through the glass plate. When the rat felt pain and withdrew its paw, the light beam turned off automatically. A cut-off time of 40 seconds was set to prevent tissue damage. Each paw of each rat was tested 5 times at intervals of 5 min. The time to respond for all paws was averaged together and used as the TWL.

### 2.5. EA Stimulation and Drug Intervention

The acupoints selected in this study were ST-36 and GB-34. The ST-36 acupoint was defined as the point 5 mm beneath the capitulum fibulae and lateral posterior to the knee joint. The GB-34 acupoint was defined as the point 6 mm below the anteroinferior head of the fibula (Figure 1(b)). Rats were fixed with an apparatus designed by our laboratory (patent application number: 201110021482.5, State Intellectual Property Office). EA treatment was applied on day 7 after CCI surgery as previously reported [24–26] and lasted between 9:00 and 11:00 a.m. daily for 7 days. EA treatment was applied using stainless steel needles which were inserted at a depth of 2-3 mm into ST36 and GB34 acupoints. The EA parameters (HANS-200E, Jisheng Medical Instruments) were set as follows: the current strength was 1.5 mA, with stimuli at alternating frequencies of 2 and 100 Hz for 30 min daily.

### 2.6. Quantitative Real-Time PCR

Total RNA isolation was extracted using the Trizol reagent (Invitrogen, Carlsbad, CA, USA). RNA (1 *μ*g) was reverse transcribed using the RNA-to-cDNA Master Mix (A3500, Promega). Real-time amplification was performed on a LightCycler 480 (Roche, USA) using SYBR Green supermix (QPK-212, Tokyo, Japan). The reaction conditions of PCR were as follows: 95°C for 10 min, followed by denaturation for 10 s at 95°C, annealing for 10 s at 60°C, and extension for 10 s at 72°C for 40 cycles. Primer sequences used for GABAAR*α*2, GABAAR*γ*2, and *β*-actin are listed in Table 1. Relative expression was calculated via the ΔΔCt method [27].

### 2.7. Western Blotting

On the 14th day after CCI, the L4–L6 spinal cord segments (1 cm) were harvested after 0.9% normal saline perfusion. The tissue samples were immediately stored at −80°C for further analysis. These tissue samples were later homogenized in cold RIPA protein lysis buffer containing PMSF (RIPA : PMSF = 100 : 1) on ice, then incubated for 30 min, and microfuged at 12,000 rpm for 30 min at 4°C, and subsequently, the supernatant was collected. The concentration of protein sample was determined using the BCA protein assay kit (Beyotime Corp, China). The homogenate was heated at 100°C for 10 min in the loading buffer. Equal amounts were loaded on 10% Tris-HCl SDS-PAGE gel (Bio-Rad Laboratories, CA), running at 80 V for approximately 30 minutes and then at 120 V for approximately 45 minutes. Following by electrophoresis, the proteins were transferred onto polyvinylidene fluoride (PVDF) (Millipore Corp, MA) membranes with a constant current of 300 mA for immunoblotting. The membranes were blocked with 5% skim milk for 2 h at room temperature and then incubated overnight at 4°C with primary antibodies: rabbit anti-GABAAR*α*2 (1 : 500; DF6626, Affinity, Cincinnati, OH, USA); rabbit anti-GABAAR*α*3 (1 : 1000; NB100-61096, Novus Biologicals, Littleton, USA); rabbit anti-GABAA-*γ*2 (1 : 1000; NB300-151, Novus Biologicals, Littleton, USA); *β*-actin (1 : 1000, #4970, Cell Signaling Technology, Beverly, MA, USA). After washing, the membranes were incubated (2 h, room temperature) with a goat anti-rabbit IgG (1 : 5000, Chemicon, USA). Protein signals were detected with a UVP gel-imaging system (Upland, CA, USA) using the WesternBright ECL reagent (Advansta, USA). The data were analyzed using AlphaEaseFC (version 4.0).

### 2.8. Immunofluorescence

After the completion of treatment, the rats were perfused intracardially with 200 ml of saline, followed by 250 ml of 4% paraformaldehyde in 0.1 M phosphate buffer (pH 7.4). Subsequently, the spinal cord segment from L4–L6 was removed and fixed in a paraformaldehyde solution. Paraffin-embedded tissue was cut into 5 *μ*m sections and mounted on poly-L-lysine-coated slides prepared for immunofluorescence staining. The sections were dewaxed and rehydrated by passage through xylene and graded ethanol. After washing, antigen retrieval was performed with citrate buffer and microwave heat induction. After three washes, the sections were incubated with 3% H_2_O_2_ for 10 min and blocked in 10% goat serum with 0.3 Triton X-100 for 1 h. Sections were incubated overnight at 4°C with a primary antibody: rabbit anti-Iba1 (microglia marker, 1 : 200, No. 019–19741, Wako, Japan); rabbit anti-GABAAR*α*2 (1 : 200); rabbit anti-GABAAR*α*3 (1 : 100, 12708-1-AP, Proteintech, China); rabbit anti-GABAAR*γ*2 (1 : 200). The next day, sections were incubated with second antibody (1 : 200, BS10950, Bioworld, MN) for 30 min at 37°C and then stained with diamidino-2-phenylindole dihydrochloride (DAPI) for 5 min. Images were taken under a fluorescence microscope (Olympus, Tokyo, Japan).

### 2.9. Transmission Electron Microscopy

The tissues were precisely cut to a size of 1 mm^3^ and fixed for 2 h with 2.5% glutaraldehyde. Subsequently, tissues were washed and subsequently fixed with osmic acid (1%) for 1 hour, dehydrated in ethanol, and embedded. The ultra-thin sections were examined with Hitachi transmission electron microscopy.

### 2.10. Statistical Analysis

Statistical analyses were carried out using SPSS 16.0 statistical software. Values for all data are expressed as means ± SD. To identify significant differences between groups, all analyses were performed by analysis of variance (ANOVA), followed by the Least Significant Difference (LSD) test. *P* values < 0.05 were considered to indicate statistical significance.

## 3. Results

### 3.1. EA Relieves Mechanical and Thermal Hyperalgesia in CCI Rats through the GABAA Receptor

We first evaluated the time course of the progression of hyperalgesia and allodynia; the TWL and MWT were examined preoperatively and at days 3, 5, 7, 10, 12, and 14 postoperatively. The five groups did not differ significantly on any of the baseline TWL and MWT measures. In the CCI + EA group, the values of TWL and MWT significantly increased on day 14 compared with those of the CCI group (*P* < 0.5). In the CCI + EA + BIC group, the MWT and TWL were worse than those in the CCI + EA group on day 14 (*P* < 0.01) (Figures 1(c) and 1(d)). These results implied that EA could relieve mechanical and thermal hyperalgesia in CCI rats, while BIC suppressed the EA-induced decrease in mechanical and thermal hyperalgesia.

### 3.2. EA Attenuates the Overexpression of Activated Microglia in the Spinal Cord of CCI Rats

**Figure 2:**
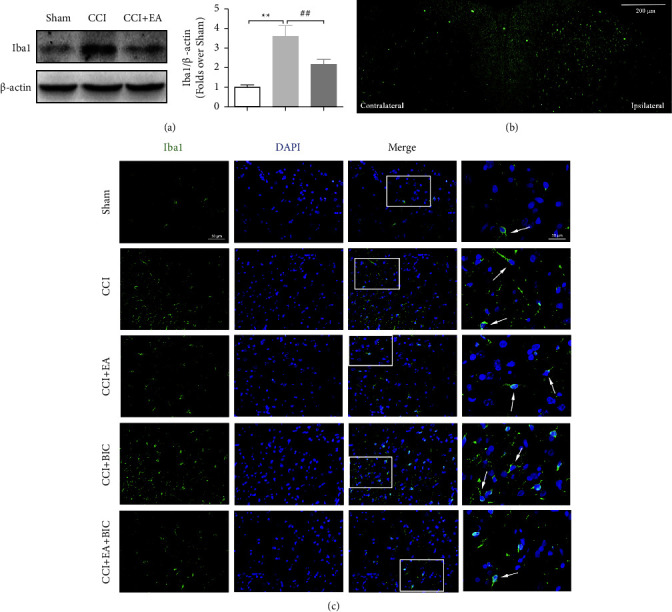
EA attenuates the overexpression of activated microglia in the spinal cord of CCI rats. (a) Western blot analysis of Iba1 (microglia marker) expression level. (b) The expression of Iba1 increased in the spinal dorsal horn ipsilateral to the nerve injury. Scale bar, 200 *μ*m. (c) Immunofluorescence staining shows Iba1 (in green) expression in the spinal dorsal horn ipsilateral to the nerve injury. Nuclei were stained with DAPI and are visualised in blue. Scale bar = 50 *μ*m, and magnification scale bar = 20 *μ*m.

To assess the role of activated microglia in the injured spinal cord, we detected the protein expression of Iba1 to investigate the effect of EA. Iba1 protein expression level was found to be significantly increased after CCI surgery; however, this increase was substantially reduced following EA treatment. In addition, immunofluorescence for activated microglia was performed in each group. Iba-1 was assessed to observe the changes in the spinal dorsal horn (Figure 2). The results revealed significant increases in the number of activated microglia in the CCI group compared with the Sham group. A decrease in the number of activated microglia was observed in the CCI + EA group relative to the CCI group. Taken together, these data suggested that EA attenuated the overexpression of activated microglia in the spinal cord.

### 3.3. EA Treatment Increases GABAA Receptor Levels in CCI Rats

**Figure 3:**
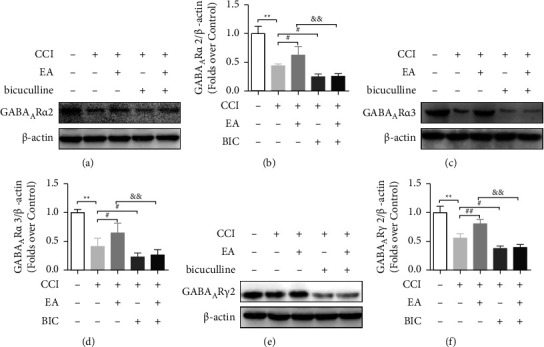
EA treatment increases protein levels of GABAAR*α*2, GABAAR*α*3, and GABAAR*γ*2 at 14 days after CCI. (a, b) Western blot analysis of GABAAR*α*2 expression level. (c, d) Western blot analysis of GABAAR*α*3 expression level. (e, f) Western blot analysis of GABAAR*γ*2 expression level. The data are presented as the means ± SD (*n* = 3–4 per group). ^*∗∗*^*P* < 0.01 compared with the Sham group; ^#^*P* < 0.05 and ^##^*P* < 0.01 compared with the CCI group; ^&&^*P* < 0.01 compared with the CCI + EA group.

**Figure 4:**
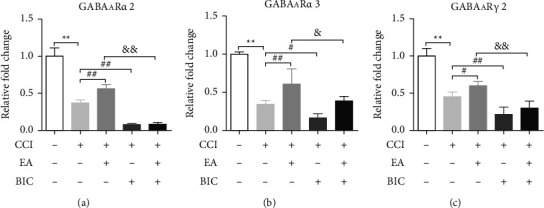
EA treatment increases mRNA levels of GABAAR*α*2 and GABAAR*γ*2 at 14 days after CCI in CCI rats. (a) GABAAR*α*2 mRNA levels. (b) GABAAR*α*3 mRNA levels. (c) GABAAR*γ*2 mRNA levels. The data are presented as the means ± SD (*n* = 3–4 per group). ^*∗∗*^*P* < 0.01 compared with the Sham group; ^#^*P* < 0.05 and ^##^*P* < 0.01 compared with the CCI group; ^&&^*P* < 0.01 compared with the CCI + EA group.

**Figure 5:**
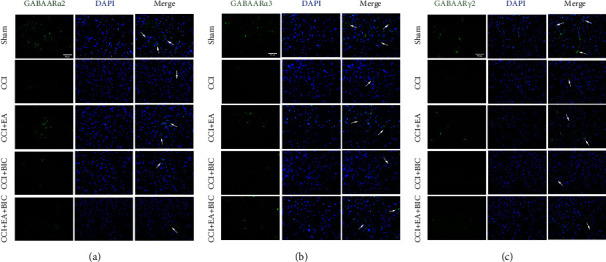
Immunofluorescence for the expression of GABAAR*α*2 and GABAAR*γ*2 in the spinal dorsal horn from rats in each group (400×). (a) Immunofluorescence staining shows GABAAR*α*2 (in green) expression in the spinal dorsal horn ipsilateral to the nerve injury. Nuclei were stained with DAPI and are visualised in blue. Scale bar = 50 *μ*m. (b) Immunofluorescence staining shows GABAAR*α*3 (in green) expression in the spinal dorsal horn ipsilateral to the nerve injury. Nuclei were stained with DAPI and are visualised in blue. Scale bar = 50 *μ*m. (c) Immunofluorescence staining shows GABAAR*γ*2 (in green) expression in the spinal dorsal horn ipsilateral to the nerve injury. Nuclei were stained with DAPI and are visualised in blue. Scale bar = 50 *μ*m.

We detected the protein expression of the GABAA receptors to further investigate the mechanism underlying the analgesic effect of EA. The Western blotting analysis showed markedly decreased levels of GABAAR*α*2, GABAAR*α*3, and GABAAR*γ*2 at 14 days after CCI (*P* < 0.01), whereas treatment with EA significantly upregulated their expression in the spinal cord (*P* < 0.05) (Figure 3). The mRNA expression of GABAAR*α*2 and GABAAR*γ*2 were detected by qRT-PCR, and the results were consistent with the Western blot results (Figure 4). Finally, we examined GABAAR*α*2 and GABAAR*γ*2 expressions by performing immunofluorescence staining. As shown in Figure 5, a marked increase in GABAAR*α*2 and GABAAR*γ*2 was observed in the ipsilateral spinal cord after EA treatment relative to CCI rats. Importantly, treatment with BIC markedly attenuated all the observed beneficial effects of EA treatment. These results showed that CCI limited GABAA receptor generation, while this can be reversed by treatment with EA.

### 3.4. EA Treatment Improved Synaptic Plasticity after CCI through the GABAA Receptor

**Figure 6:**
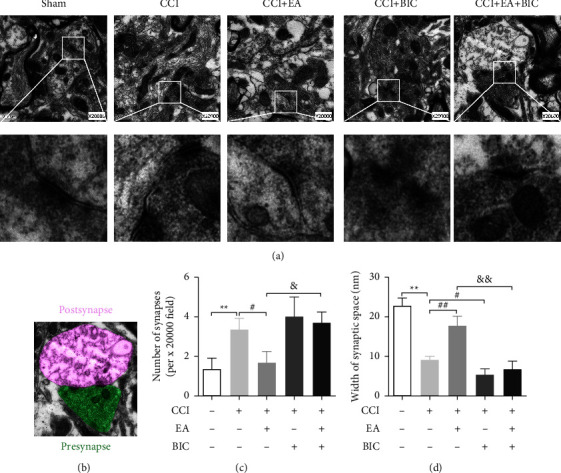
The ultrastructure of synapses in the spinal dorsal horns of all groups. (a) Ultrastructures of the synapses in the spinal dorsal horn ipsilateral to the nerve injury using TEM. Scale bar = 0.5 *μ*m. (b) Schematic of the presynaptic (green) and postsynaptic (violet) structures. (c) The number of synapses in the spinal dorsal horn in each group. (d) Width of the synaptic space (nm). The data are presented as the means ± SD (*n* = 3 per group). ^*∗∗*^*P* < 0.01 compared with the Sham group; ^#^*P* < 0.05 and ^##^*P* < 0.01 compared with the CCI group; ^&^*P* < 0.05 and ^&&^*P* < 0.01 compared with the CCI + EA group.

We used transmission electron microscopy to examine the morphological changes of the synapses in each group. As shown in Figure 6(a), the synaptic structure in the Sham group was clear, with presynaptic and postsynaptic membranes and synaptic cleft. Compared with the Sham group, the CCI group exhibited abnormal synapses, including vague presynaptic membranes, thicker postsynaptic densities, and narrow synaptic clefts. The number and morphology of the synapses in the EA group were far more regular than in the CCI group, as shown in Figure 6(c). The synapses of the CCI + EA + BIC group appeared to have narrower synaptic clefts and more synapses than those of the CCI + EA group (Figure 6(d)). These results suggested that hypersensitivity reaction occurred after modeling, abnormal synapses increased, and synaptic clefts became narrower. This phenomenon can be reversed by treatment with EA, while BIC suppressed the effect of EA.

## 4. Discussion

Neuropathic pain is a growing public health concern across the globe. Investigations over the last few decades have greatly improved our knowledge on the mechanism of neuropathic pain. Currently, there is no effective therapy for neuropathic pain, largely because of the complex nature of this disease. In this study, using a CCI-induced neuropathic pain model, we found that repeated EA at ST-36 and GB-34 has a beneficial analgesic effect on chronic neuropathic pain and the mechanical and thermal nociceptive thresholds were elevated. In addition, our data suggested that EA treatment could inhibit the activation of microglia and the upregulation of GABAA receptors.

Microglia are the primary immune cells in the CNS. Under normal conditions, they are in a resting state, having a small soma with thin and branched processes, and are activated when pathological stimulation occurs [28, 29]. Blocking microglial activation in spinal cord microglia could suppress the development and maintenance of hyperalgesia and allodynia after neuropathic pain occurs. Activated microglia upregulated their surface expression of immunomodulatory proteins and became efficient in releasing neurotransmitters and immunomodulators [30]. The transmission of pain signals from periphery to central sensitization is a dynamic process [31]. Pain signals could be amplified by the transmission of nociceptive information (hyperalgesia), suppressed (analgesia), or relayed unaltered [32]. Pain modulation occurred in the dorsal horn of the spinal cord, where peripheral nerves relay sensory information to the brain through projection neurons [33]. Currently, pain hyperalgesia in the CNS is thought to be triggered by a series of neuronal events in the dorsal horn of the spinal cord [34]. Available experimental evidence indicates that excessive microglial activation in the dorsal horn is involved in the development of neuropathic pain or pain-related behavior. As the microglia have been widely accepted for their roles in neuropathic pain, an immunofluorescence assay was used in this study to detect the activation of microglia. The activated microglia, in large numbers, were observed in the ipsilateral spinal cord of rats subjected to CCI surgery. Microglia play a key role in promoting pain states through several known mechanisms. The activation of microglia was strongly associated with the decrease in KCC2 expression and less hyperpolarizing GABA signaling. The downregulation of KCC2 mediated by activated microglia leads to an increase in intracellular chloride ions and a decrease in inhibitory input, thereby reducing inhibitory activity and promoting neuropathic pain [35]. In the previous study, we found that EA treatment has an effect on the BDNF-TrkB pathway [36]. In this study, we further explored the effect of EA on GABAA receptor subunits, which are downstream of KCC2 to confirm the important role of GABAAR in EA analgesia. GABAA receptors are dynamically regulated and can be rapidly internalized leading to remove from the surface of neurons. In the past few decades, many laboratories have made great efforts to elucidate the mechanisms by which BDNF is synthesized and released by microglia after the injury occurs, the downstream TrkB-mediated signaling in neurons, and the biophysical mechanism of inhibition is relieved by the regulation of the KCC2, a postsynaptically restricted Cl−/K+ cotransporter that functions to regulate chloride homeostasis in neurons and is essential for GABAA receptor-mediated depolarization [37]. The GABAergic signaling pathway is indispensable for the coordination of the perception and response of pain circuits in the CNS [38, 39]. In the present work, we demonstrate that GABAA receptor antagonists significantly aggravate neuropathic pain.

EA has been suggested as a therapeutic intervention for chronic pain. EA is widely used in treating pain diseases and has good clinical effects, several studies have shown that EA has an immunomodulatory effect [40, 41], and we hypothesized that the EA analgesic effect might be associated with its regulation of the spinal microglia and GABAA receptors. In addition, experimental studies and clinical observations suggested that the EA signal pathway is intricately entwined with the pain pathways, converging and integrating into the dorsal horn of the spinal cord and medial thalamus. It has been proposed that BDNF regulates the neural circuit through binding with its receptor TrkB, and the release and information transduction mechanism controlled by electrical stimulation is one of the possible mechanisms of acupuncture [42]. A recent study indicated that repeated EA stimulation at ST36 and GB34 acupoints could alleviate the sensory and emotional aspects of chronic pain in chronic pain-negative rats, which may be related to the upregulation of GABAA*β*2, GABAB1, NMDA-NR1, PSD-95, and Piccolo genes and promoting the expression of mGluR1 and GABAB2 proteins and Piccolo gene in the amygdala [43]. Another study showed that EA at LI18, LI4, and PC6 could alleviate incisional neck pain, which may be related to its effect on upregulating GABA expression in the cervical (C3-6) dorsal root ganglions [44]. Although a large number of reports have proved that spinal cord TrkB receptor, KCC2, and GABAA receptor participate in neuropathic pain, the research on the effect of EA on the above indicators in the neuropathic pain model is still very scarce. It is hypothesized that BDNF synthesis is increased by activated microglia in the spinal cord after peripheral nerve injury and BDNF inhibits KCC2 expression after binding to TrkB receptors in the spinal cord. However, whether the GABAA receptor subsets in the L4-6 spinal cord are involved in EA analgesia remains unclear. In this study, we found that the microglia were interestingly increased and GABAA receptors were decreased in the spinal dorsal horn of CCI rats. Previous studies have shown that neuropathic pain causes peripheral ectopic pulses to remain excitatory for a long time, resulting in changes in synaptic morphology, including narrowing of synaptic cleats, increase of synaptic interface curvature, and increase of synaptic vesicles [45, 46]. These changes in synaptic morphology may result in aberrant signaling between neurons. Our results were consistent with previous studies; hypersensitivity reaction occurred after modeling, abnormal synapses increased, and synaptic clefts became narrower. This phenomenon was reversed after EA treatment, while BIC suppressed the effect of EA.

In summary, our data suggest that the inhibition of microglial activation and upregulation of GABAA receptor expression might account for the analgesic mechanisms. EA significantly alleviated CCI-induced microglial activation and upregulated GABAA receptor expressions, and it was effective at alleviating hyperalgesia in CCI rats. EA may be a potential treatment method for neuropathic pain. Hence, our study provides evidence for the possible therapeutic benefit of EA to treat chronic neuropathic pain. Multiple mechanisms involving several biological processes and biochemistries may be involved in CCI-induced neuropathic pain relief; further studies would be needed to explore the mechanisms underlying the effect of EA treatment.

## Figures and Tables

**Table 1:** Primers sequence for quantitative real-time polymerase chain reaction.

Gene	Forward primer (5′-3′)	Reverse primer (5′-3′)
GABAAR*α*2	ACAGTCCAAGCCGAATGTCCAATG	CTTCCGAGGTCGTGTAAGCATAGC
GABAAR*α*3	CACCACTGTTCTCACCATGACCAC	CAGACGGCTATGAACCAGTCCATG
GABAAR*γ*2	TTTGGATGGCAAGGACTG	AGAAGGCGGTAGGGAAGA
*β*-Actin	TGGCTCTATCCTGGCCTCAC	CGCAGCTCAGTAACAGTCCG

## Data Availability

The analyzed data sets generated during the study are available from the corresponding author upon reasonable request.
